# *mir-355* Functions as An Important Link between p38 MAPK Signaling and Insulin Signaling in the Regulation of Innate Immunity

**DOI:** 10.1038/s41598-017-15271-2

**Published:** 2017-11-06

**Authors:** Lingtong Zhi, Yonglin Yu, Zhixia Jiang, Dayong Wang

**Affiliations:** Key Laboratory of Developmental Genes and Human Diseases in Ministry of Education, Medical School, Southeast University, Nanjing, 210009 China

## Abstract

We performed a systematic identification of microRNAs (miRNAs) involved in the control of innate immunity. We identified 7 novel miRNA mutants with altered survival, colony forming in the body, and expression pattern of putative antimicrobial genes after *Pseudomonas aeruginosa* infection. Loss-of-function mutation of *mir-45, mir-75, mir-246, mir-256*, or *mir-355* induced resistance to *P. aeruginosa* infection, whereas loss-of-function mutation of *mir-63* or *mir-360* induced susceptibility to *P. aeruginosa* infection. DAF-2 in the insulin signaling pathway acted as a target for intestinal *mir-355* to regulate innate immunity. *mir-355* functioned as an important link between p38 MAPK signaling pathway and insulin signaling pathway in the regulation of innate immunity. Our results provide an important molecular basis for further elucidation of the functions of various miRNAs in the regulation of innate immunity.

## Introduction

microRNAs (miRNAs), a class of non-coding RNAs with 19–22 nucleotides, are encoded within the genome in organisms^1^. miRNAs are initially transcribed as primary transcripts (pri-miRs). The pri-miRs are further cleaved to produce 70 nucleotide-long precursor miRNAs (pre-miRs) and then mature miRNAs, respectively^1^. The mature miRNAs regulate various fundamental biological processes by imperfectly binding their multiple targeted mRNAs and suppressing the expression of their targeted genes post-transcriptionally^2,3^. Bioinformatic or functional analyses has suggested that miRNAs can directly target multiple proteins, implying the property of multiple functions for miRNAs^4^. *Caenorhabditis elegans* is a powerful model animal to determine the functions and mechanisms of miRNAs in regulating certain biological processes, such as transition of developmental timing and longevity^5–7^. For example, *lin-4* and *let-7* have been proven to be involved in the control of transition of developmental timing^8,9^. *lin-4* and *let-7*, two important founding members of miRNAs, were first identified in *C. elegans via* forward genetic screens^8,9^.

*C. elegans* is also a wonderful model for the study of innate immune response to pathogen infection or host-pathogen interactions, because its intestine consisting of 20 epithelial cells is full of microbes^10,11^. In *C. elegans*, once certain pathogenic bacteria are accumulated in the intestine, they will invade the host cells and even kill the animals during infectious processes^12^. Upon infection, *C. elegans* can potentially avoid the pathogens or activate an inducible innate immune system^13^. Innate immunity plays a pivotal role in being against pathogen infection in animal kingdom, and *C. elegans* can provide mechanistic insights into conserved signal transduction of innate immunity and host-pathogen interactions^13,14^. Some important and conserved signaling pathways, including p38 mitogen-activated protein kinase (MAPK), insulin, and TGF-β signaling pathways, have been identified to be required for the control of innate immunity in *C. elegans* ^15–17^. Recently, some miRNAs, such as *let-7, mir-84*, *mir-241*, *mir-251*, *mir-252*, and *mir-2*33, have been further shown to be involved in the control of innate immune response to pathogen infection in *C. elegans* ^18–21^. Nevertheless, the potential involvement of most of miRNAs in the control of innate immunity is still unknown in *C. elegans*.

*Pseudomonas aeruginosa* is considered to be toxic, and can cause a lethal intestinal infection on nematode host^22,23^. Upon early *P. aeruginosa* infection, *C. elegans* can upregulate mRNA expression of some defense genes, including genes encoding anti-microbial peptides^22^. In the present study, we performed a systematic identification of the possible miRNAs involved in the control of innate immune response to *P. aeruginosa* PA14 infection in *C. elegans*. Moreover, we focused on *mir-*3*55* to examine its molecular basis in the regulation of innate immunity. Our results provide an important basis for further understanding and systematically elucidating the functions of miRNAs in the regulation of innate immunity.

## Results

### Mutations of some miRNAs altered the survival of nematodes infected with *P. aeruginosa* PA14

**Figure 1:**
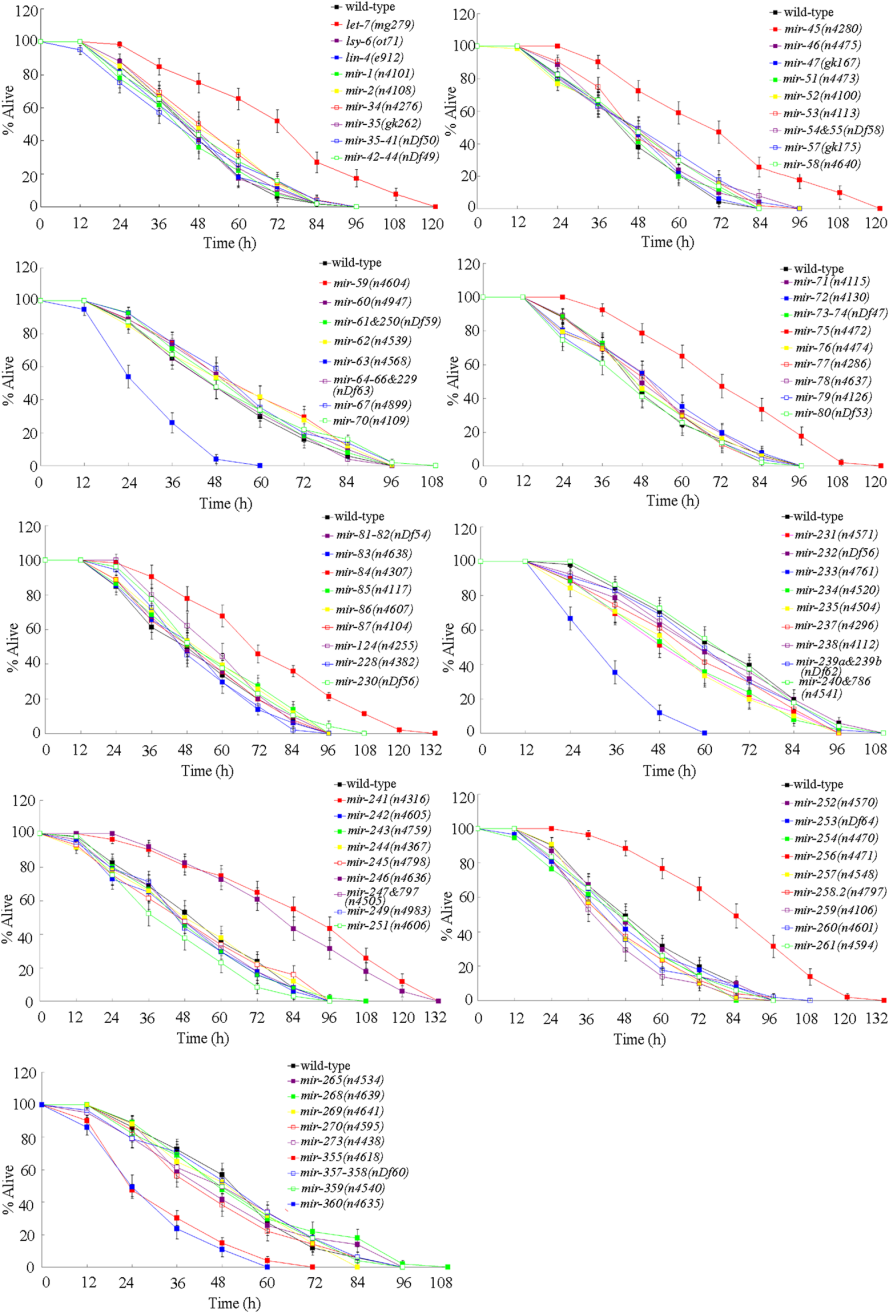
Survival in miRNA mutants infected with *P. aeruginosa* PA14. Bars represent mean ± SD.

Using deletion mutants, we performed a systematic identification of miRNAs involved in the control of *P. aeruginosa* PA14 infection and the corresponding innate immune response in nematodes. Based on phenotypic analysis of survival in miRNA mutants infected with *P. aeruginosa* PA14, we identified 11 miRNA mutants out of the examined 82 miRNA mutants with the abnormal survival compared with wild-type nematodes (Fig. 1, Table S1). These miRNA mutants were *let-7(mg279)*, *mir-45(n4280)*, *mir-6*3*(n4568)*, *mir-75(n4472)*, *mir-84(n4*3*07)*, *mir-2*33*(n4761)*, *mir-241(n4316)*, *mir-246(n4636)*, *mir-256(n4471)*, *mir-355(n4618)*, and *mir-360(n4635)* (Fig. 1). Loss-of-function mutation of *let-7*, *mir-45*, *mir-75*, *mir-84*, *mir-241*, *mir-246*, or *mir-256* caused the resistance to the adverse effect of *P. aeruginosa* PA14 infection on survival in nematodes (Fig. 1). In contrast, loss-of-function mutation of *mir-63*, *mir-233*, *mir-360*, or *mir-355* resulted in the susceptibility to the adverse effect of *P. aeruginosa* PA14 infection on survival in nematodes (Fig. 1). Statistical comparisons of the survival plots demonstrated that, after *P. aeruginosa* PA14 infection, the survival of *let-7(mg279)*, *mir-45(n4280)*, *mir-63(n4568)*, *mir-75(n4472)*, *mir-84(n4307)*, *mir-233(n4761)*, *mir-241(n4316)*, *mir-246(n4636)*, *mir-256(n4471)*, *mir-355(n4618)*, or *mir-360(n4635)* was significantly (*P* < 0.001) different from that of wild-type nematodes (Table S1). Among these 11 candidate miRNA mutants, *let-7(mg279), mir-84(n4307)*, *mir-241(n4316)*, and *mir-233(n4761)* mutants have been reported in the previous studies^18–20,22^. We next examined the *P. aeruginosa* PA14 colony-forming unit (CFU) and the expression pattern of putative antimicrobial genes in the other 7 miRNA mutants infected with *P. aeruginosa* PA14.

### *P. aeruginosa* PA14 CFU in the new identified miRNA mutants after infection

**Figure 2:**
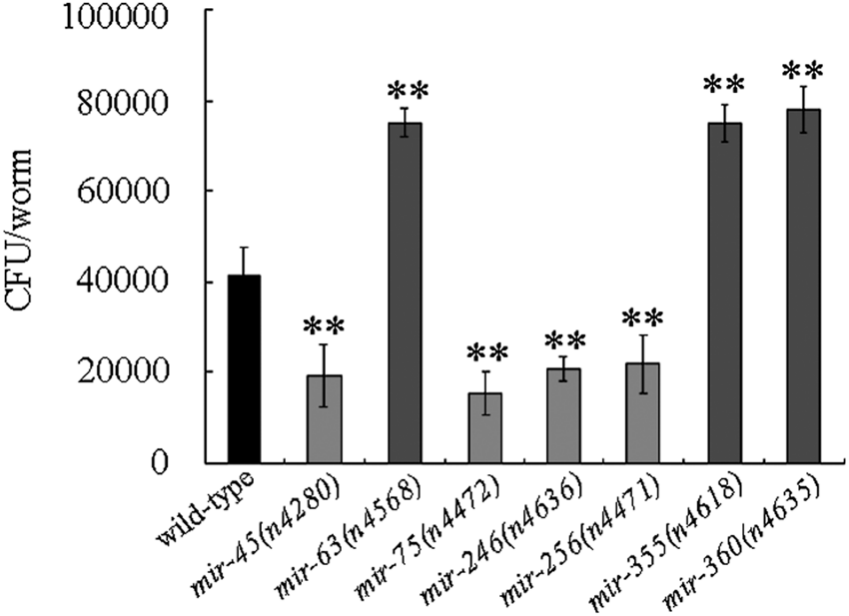
*P. aeruginosa* PA14 CFU in the body of miRNA mutants infected with *P. aeruginosa* PA14. Bars represent mean ± SD. **P < 0.01 *vs* wild-type.

We employed the CFU to determine PA14 colony formation in the body of miRNA mutant after *P. aeruginosa* infection. After *P. aeruginosa* PA14 infection, we observed that loss-of-function mutation of *mir-63*, *mir-360*, or *mir-355* significantly enhanced the PA14 colony formation in the body of nematodes (Fig. 2). Different from these, after *P. aeruginosa* PA14 infection, loss-of-function mutation of *mir-45*, *mir-75*, *mir-246*, or *mir-256* significantly suppressed the PA14 colony formation in the body of nematodes (Fig. 2).

### Expression patterns of putative antimicrobial genes in the new identified miRNA mutants after *P. aeruginosa* infection

**Figure 3:**
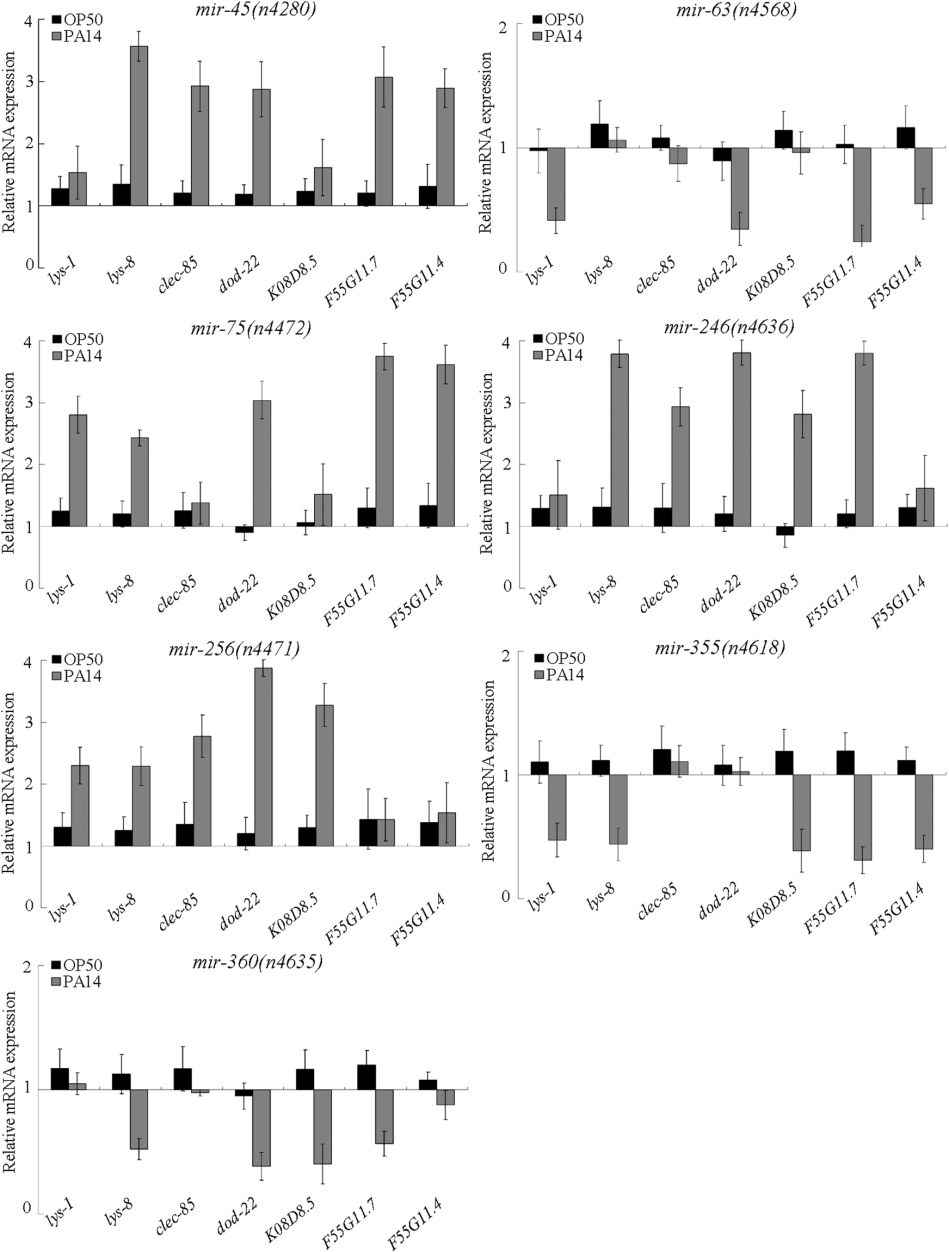
Expression patterns of putative antimicrobial genes in *P. aeruginosa* PA14 infected miRNA mutant nematodes. Normalized expression is presented relative to wild-type expression. Bars represent mean ± SD.

We selected some putative antimicrobial genes (*lys-1*, *lys-8*, *clec-85*, *dod-22*, *K08D8.5*, *F55G11.7*, and *F55G11.4*) to further determine the innate immune response in *P. aeruginosa* PA14 infected miRNA mutants. *P. aeruginosa* PA14 infection significantly increases the transcriptional expression of these antimicrobial genes^14^. In *C. elegans*, *lys-1* and *lys-8* encode lysozymes, *clec-85* encodes a C-type lectin protein, *dod-22* and *F55G11.7* encode orthologs of human epoxide hydrolase 1, and *K08D8.5* and *F55G11.4* encode CUB-like domain-containing proteins. After *P. aeruginosa* PA14 infection, mutation of *mir-45* increased the expression levels of *lys-8*, *clec-85*, *dod-22*, *F55G11.7*, and *F55G11.4*, mutation of *mir-75* increased the expression levels of *lys-1*, *lys-8*, *dod-22*, *F55G11.7*, and *F55G11.4*, mutation of *mir-246* increased the expression levels of *lys-8*, *clec-85*, *dod-22*, *K08D8.5*, and *F55G11.7*, and mutation of *mir-256* increased the expression levels of *lys-1*, *lys-8*, *clec-85*, *dod-22*, and *K08D8.5* (Fig. 3). In contrast, mutation of *mir-63* decreased the expression levels of *lys-1*, *dod-22*, *F55G11.7*, and *F55G11.4*, mutation of *mir-355* decreased the expression levels of *lys-1*, *lys-8*, *K08D8.5*, *F55G11.7*, and *F55G11.4*, and mutation of *mir-360* decreased the expression levels of *lys-8*, *dod-22*, *K08D8.5*, and *F55G11.7* (Fig. 3). Therefore, loss-of-function mutation of these 7 miRNAs may alter the innate immune response of nematodes to *P. aeruginosa* PA14 infection.

### Prediction of targets for new identified miRNAs during the control of innate immune response to *P. aeruginosa* PA14 infection

We further used TargetScan software (http://www.targetscan.org/worm_52/) with preferentially conserved targeting (PCT) between 0 and 1 and miRBase (http://www.mirbase.org) with a score threshold of −0.1 to predict potential targets for new identified miRNAs in regulating the innate immune response by searching for the presence of conserved sites that match the seed region of new identified miRNAs^24,25^. In *C. elegans*, insulin and TGF-β signaling pathways are two important signaling pathways in the control of innate immune response to *P. aeruginosa* PA14 infection^16,17^. In the insulin signaling pathway, *daf-2* gene encodes an insulin receptor. In the TGF-β signaling pathway, *sma-3* gene encodes a Smad protein. Among the predicted targets, we found that SMA-3 in the TGF-β signaling pathway might function as the potential target for *mir-246* in the regulation of innate immunity, and DAF-2 in the insulin signaling pathway might function as the potential target for *mir-355* in the regulation of innate immunity. We next focused on the *mir-355* to examine its molecular basis in the regulation of innate immune response to *P. aeruginosa* PA14 infection. In *C. elegans*, after *P. aeruginosa* PA14 infection, we observed the significant increase in the *mir-355* expression (Fig. S1).

### Genetic interaction between *mir-355* and DAF-2 in the regulation of innate immune response to *P. aeruginosa* PA14 infection

**Figure 4:**
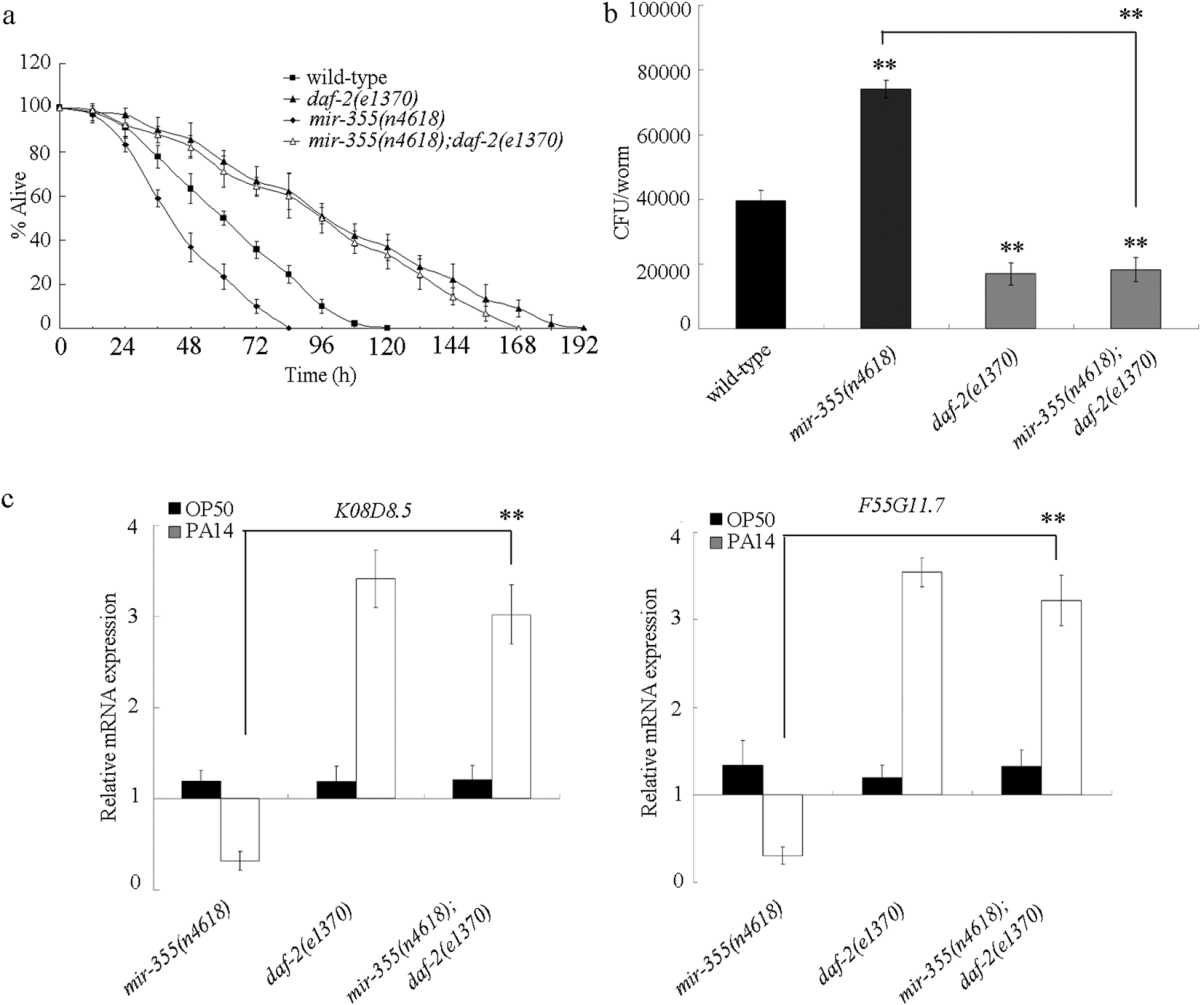
Genetic interaction between *mir-355* and DAF-2 in the regulation of innate immune response to *P. aeruginosa* PA14 infection. (**a**) Genetic interaction between *mir-355* and DAF-2 in the regulation of survival in *P. aeruginosa* PA14 infected nematodes. The survival was analyzed at 20 °C. Statistical comparisons of the survival plots indicate that, after *P. aeruginosa* PA14 infection, the survival of *mir-355(n4618);daf-2(e1370)* was significantly different from that of *mir-355(n4618)* (*P* < 0.001). Bars represent mean ± SD. (**b**) Genetic interaction between *mir-355* and DAF-2 in the regulation of *P. aeruginosa* PA14 CFU in the body of nematodes. Bars represent mean ± SD. **P < 0.01 *vs* wild-type (if not specially indicated). (**c**) Genetic interaction between *mir-355* and DAF-2 in the regulation of expression patterns of putative antimicrobial genes in *P. aeruginosa* PA14 infected nematodes. Normalized expression is presented relative to wild-type expression. Bars represent mean ± SD. **P < 0.01.

We assumed that the *daf-2* mutation would suppress the phenotypes in nematodes with *mir-355* mutation, if DAF-2 is the target of *mir-355*. After *P. aeruginosa* PA14 infection, mutation of *daf-2* significantly increased the survival, decreased the *P. aeruginosa* PA14 CFU, and enhanced the expression levels of putative antimicrobial genes (*K08D8.5* and *F55G11.7*)^22^ in *mir-355(n4618)* mutant (Fig. 4). Therefore, DAF-2 may be the target for *mir-355* in the regulation of innate immune response to *P. aeruginosa* PA14 infection.

### Effects of intestinal overexpression of *daf-2* lacking 3′ UTR or containing 3′ UTR on innate immune response of nematodes overexpressing intestinal *mir-355* to *P. aeruginosa* PA14 infection

**Figure 5:**
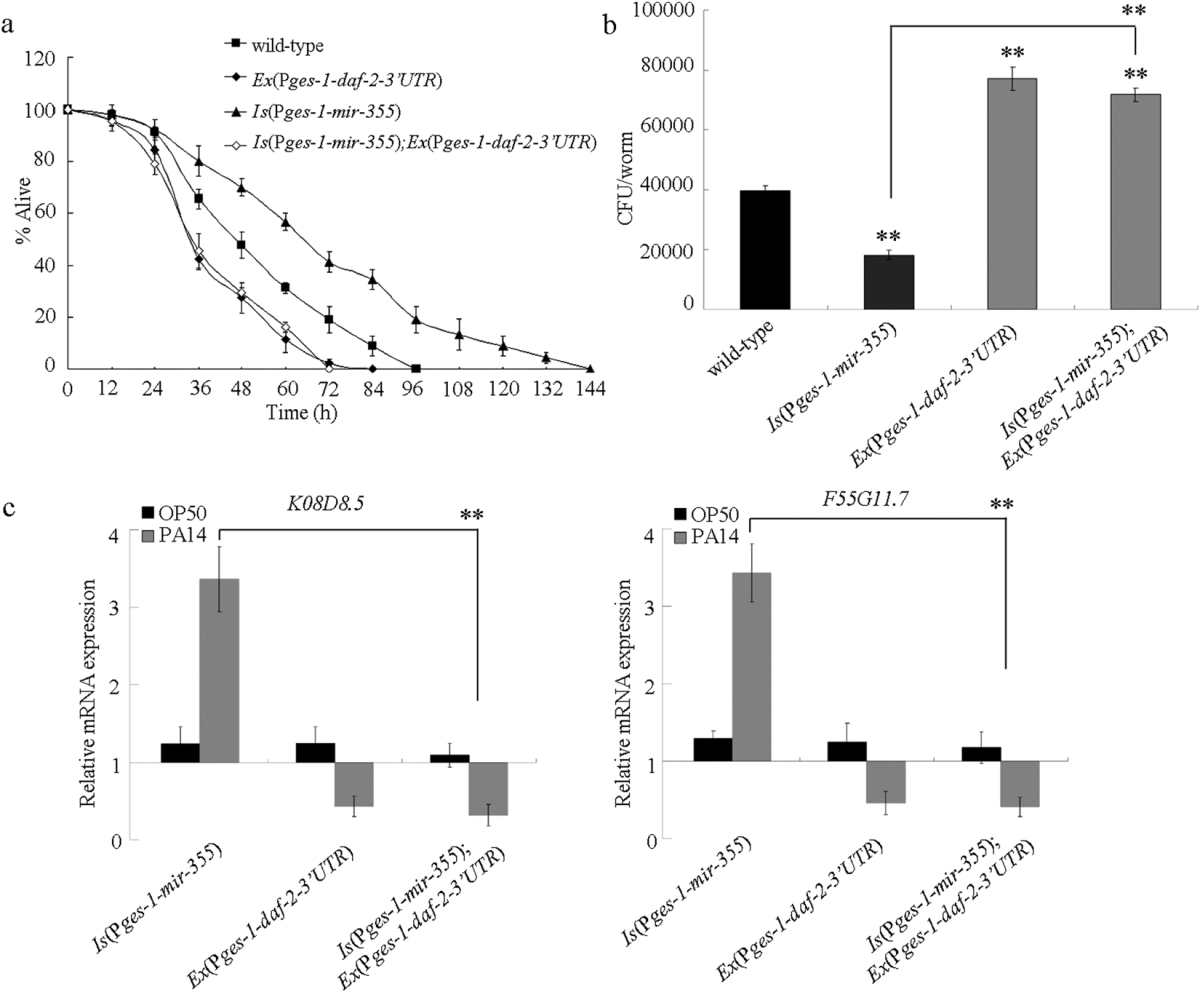
Effects of intestinal overexpression of *daf-2* lacking 3′ UTR on innate immune response to *P. aeruginosa* PA14 infection in nematodes overexpressing intestinal *mir-355*. (**a**) Effects of intestinal overexpression of *daf-2* lacking 3′ UTR on survival of nematodes overexpressing intestinal *mir-355* after *P. aeruginosa* PA14 infection. Statistical comparisons of the survival plots indicate that, after the *P. aeruginosa* PA14 infection, the survival of transgenic strain *Is(*P*ges-1-mir-355);Ex(*P*ges-1-daf-2-3*′*UTR)* was significantly different from that of transgenic strain *Is(*P*ges-1-mir-355)* (*P* < 0.001). Bars represent mean ± SD. (**b**) Effects of intestinal overexpression of *daf-2* lacking 3′ UTR on *P. aeruginosa* PA14 CFU in the body of nematodes overexpressing intestinal *mir-355*. Bars represent mean ± SD. **P < 0.01 *vs* wild-type (if not specially indicated). (**c**) Effects of intestinal overexpression of *daf-2* lacking 3′ UTR on expression patterns of putative antimicrobial genes of nematodes overexpressing intestinal *mir-355* after *P. aeruginosa* PA14 infection. Normalized expression is presented relative to wild-type expression. Bars represent mean ± SD. **P < 0.01.

In *C. elegans*, *mir-355* is expressed in the intestine^26^. Meanwhile, the insulin signaling pathway can function in the intestine to regulate the innate immunity in nematodes^27^. To further confirm the role of DAF-2 as a molecular target of intestinal *mir-355* in the regulation of innate immunity, we introduced the intestinal *daf-2* lacking 3′ UTR (*Ex(*P*ges-1-daf-2-3*′*UTR)*) into the transgenic nematodes overexpressing intestinal *mir-355*. After *P. aeruginosa* PA14 infection, the transgenic strain *Is(*P*ges-1-mir-355);Ex(*P*ges-1-daf-2-3*′*UTR)* exhibited the similar survival to that in the transgenic strain *Ex*(P*ges-1-daf-2-3*′*UTR)* (Fig. 5a). The *P. aeruginosa* PA14 CFU in the transgenic strain *Is(*P*ges-1-mir-355);Ex(*P*ges-1-daf-2-3*′*UTR)* was similar to that in the transgenic strain *Ex*(P*ges-1-daf-2-3*′*UTR)* (Fig. 5b). Moreover, the expression patterns of putative antimicrobial genes (*K08D8.5* and *F55G11.7*) in the transgenic strain *Is(*P*ges-1-mir-355);Ex(*P*ges-1-daf-2-3*′*UTR)* were similar to those in the transgenic strain *Ex*(P*ges-1-daf-2-3*′*UTR)* (Fig. 5c). Therefore, intestinal overexpression of *daf-2* lacking 3′ UTR may effectively suppress the resistance of nematodes overexpressing intestinal *mir-355* to *P. aeruginosa* PA14 infection.

**Figure 6:**
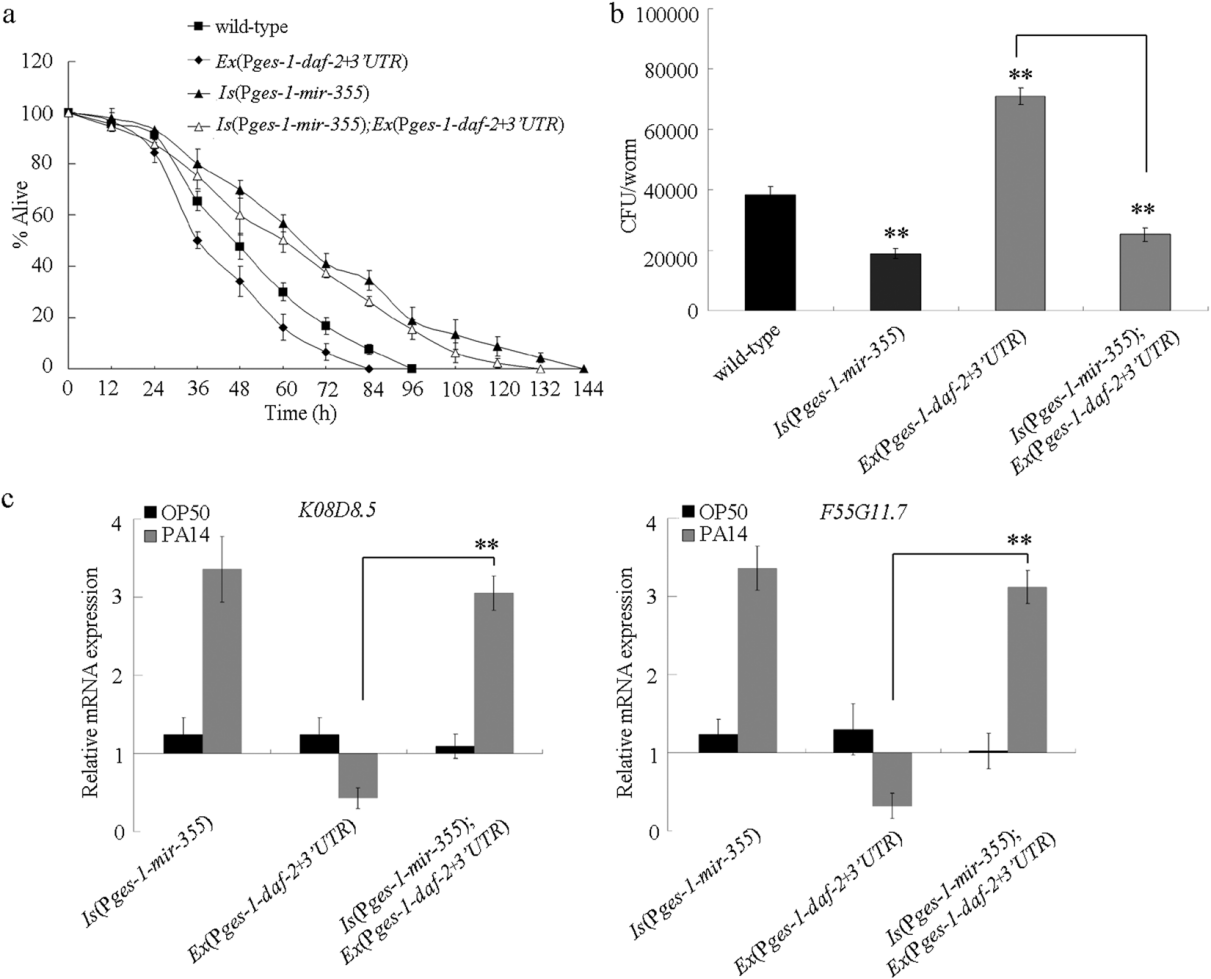
Effects of intestinal overexpression of *daf-2* containing 3′ UTR on innate immune response to *P. aeruginosa* PA14 infection in nematodes overexpressing intestinal *mir-355*. (**a**) Effects of intestinal overexpression of *daf-2* containing 3′ UTR on survival of nematodes overexpressing intestinal *mir-355* after *P. aeruginosa* PA14 infection. Statistical comparisons of the survival plots indicate that, after the *P. aeruginosa* PA14 infection, the survival of transgenic strain *Is(*P*ges-1-mir-355);Ex(*P*ges-1-daf-2* + *3*′*UTR)* was significantly different from that of transgenic strain of *Ex(*P*ges-1-daf-2* + *3*′*UTR)* (*P* < 0.001). Bars represent mean ± SD. (**b**) Effects of intestinal overexpression of *daf-2* containing 3′ UTR on *P. aeruginosa* PA14 CFU in the body of nematodes overexpressing intestinal *mir-355*. Bars represent mean ± SD. **P < 0.01 *vs* wild-type (if not specially indicated). (**c**) Effects of intestinal overexpression of *daf-2* containing 3′ UTR on expression patterns of putative antimicrobial genes of nematodes overexpressing intestinal *mir-355* after *P. aeruginosa* PA14 infection. Normalized expression is presented relative to wild-type expression. Bars represent mean ± SD. **P < 0.01.

We also introduced the intestinal *daf-2* containing the 3′ UTR (*Ex(*P*ges-1-daf-2* + *3*′*UTR)*) into the transgenic nematodes overexpressing intestinal *mir-355*. After *P. aeruginosa* PA14 infection, the transgenic strain *Is(*P*ges-1-mir-355);Ex(*P*ges-1-daf-2* + *3*′*UTR)* exhibited the similar survival to that in the transgenic strain *Is(*P*ges-1-mir-355)* (Fig. 6a). The *P. aeruginosa* PA14 CFU in the transgenic strain *Is(*P*ges-1-mir-355);Ex(*P*ges-1* + *daf-2* + *3*′*UTR)* was also similar to that in the transgenic strain *Is(*P*ges-1-mir-355)* (Fig. 6b). Moreover, we observed that the expression patterns of antimicrobial genes (*K08D8.5* and *F55G11.7*) in the transgenic strain *Is(*P*ges-1-mir-355);Ex(*P*ges-1-daf-2* + *3*′*UTR)* were similar to those in the transgenic strain *Is(*P*ges-1-mir-355)* (Fig. 6c). These results suggest that intestinal overexpression of *mir-355* can inhibit the susceptibility of nematodes overexpressing intestinal *daf-2* containing 3′ UTR.

### *In vivo* 3′-UTR binding assay of *daf-2*

To further confirm whether *mir-355* regulated the protein levels of DAF-2 through 3′-UTR, we generated a *ges-1* promoter driven GFP vector containing 3′-UTR of *daf-2(*P*ges-1::GFP-3*′*-UTR) (daf-2 wt)* or P*ges-1::GFP-3*′*-UTR (daf-2 mut)*. A *daf-2 3*′*-UTR* mutant reporter construct was generated by replacing the putative *mir-355* binding site with an oligonucleotide containing the exact identical sequence of *mir-355*. A *Pges-1::mCherry3*′*-UTR(tag-196)* construct that drives the mCherry expression was employed as an internal control. After *P. aeruginosa* PA14 infection, the GFP expression was suppressed in wild-type nematodes (Fig. S2). In contrast, mutagenesis of putative binding site for *mir-355* in *daf-2 3*′*-UTR* abolished this suppression of GFP expression in wild-type nematodes (Fig. S2). After *P. aeruginosa* PA14 infection, we observed the higher GFP expression in *mir-355(n4618)* mutant than that in wild-type nematodes (Fig. S2). These results demonstrate that *mir-355* may inhibit the DAF-2 function through binding to its 3′-UTR and suppressing its translation in *P. aeruginosa* PA14 infected nematodes.

### *mir-355* acted downstream of PMK-1 to regulate the innate immune response to *P. aeruginosa* PA14 infection

**Figure 7:**
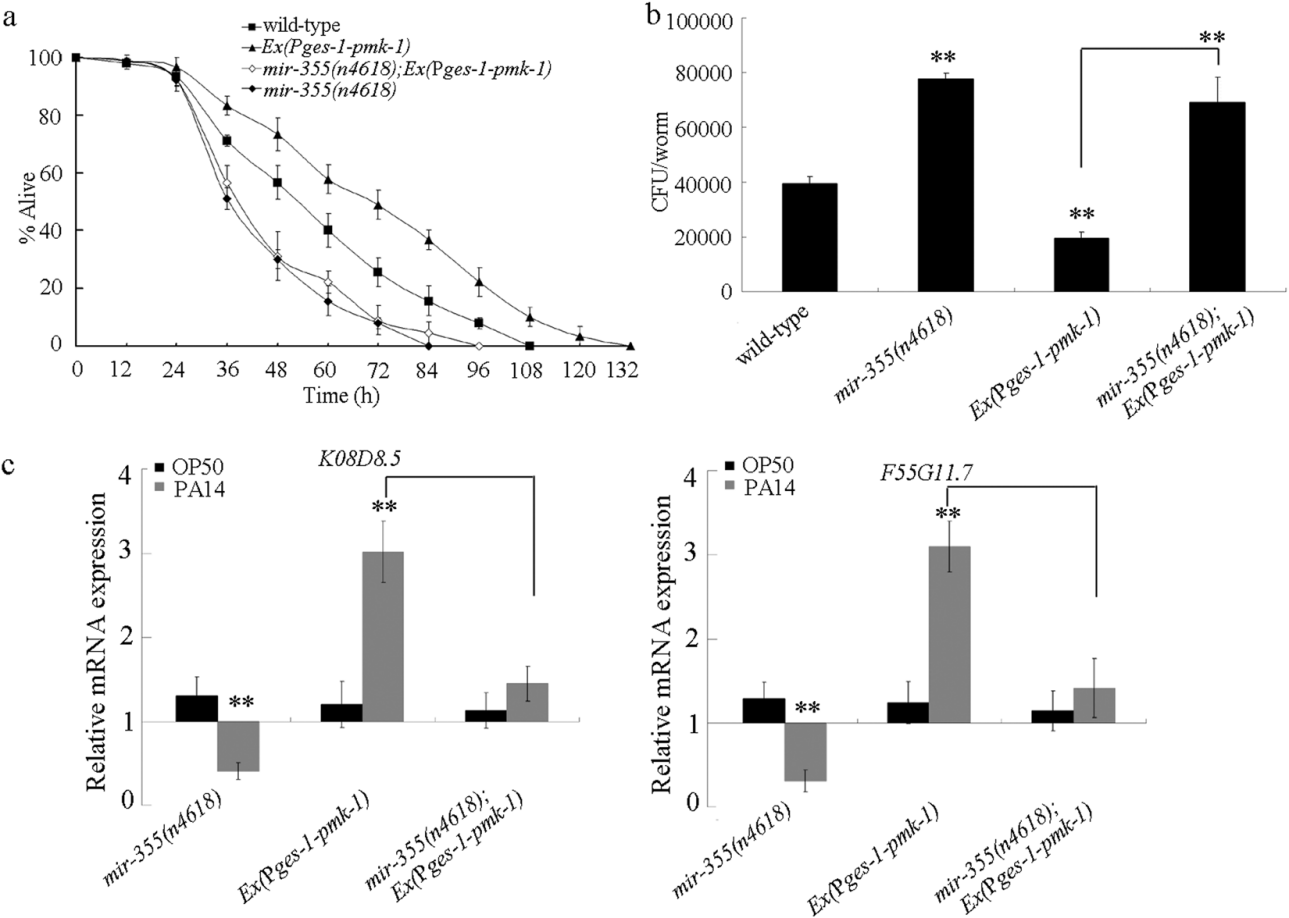
Genetic interaction between *mir-355* and PMK-1 in the regulation of innate immune response to *P. aeruginosa* PA14 infection. (**a**) Genetic interaction between *mir-355* and PMK-1 in the regulation of survival in *P. aeruginosa* PA14 infected nematodes. Statistical comparisons of the survival plots indicate that, after *P. aeruginosa* PA14 infection, the survival of *mir-355(n4618);Ex(*P*ges-1-pmk-1)* was significantly different from that of *Ex(*P*ges-1-pmk-1)* (*P* < 0.001). Bars represent mean ± SD. (**b**) Genetic interaction between *mir-355* and PMK-1 in the regulation of *P. aeruginosa* PA14 CFU in the body of nematodes. Bars represent mean ± SD. **P < 0.01 *vs* wild-type (if not specially indicated). (**c**) Genetic interaction between *mir-355* and PMK-1 in the regulation of expression patterns of putative antimicrobial genes in *P. aeruginosa* PA14 infected nematodes. Normalized expression is presented relative to wild-type expression. Bars represent mean ± SD. **P < 0.01.

In *C. elegans*, p38 MAPK signaling pathway is a conserved signaling pathway required for the pathogen resistance^13,15^. In the p38 MAPK signaling pathway, *pmk-1* encodes a p38 MAPK. Overexpression of intestinal *pmk-1* induced a resistance to *P. aeruginosa* PA14 infection, decreased *P. aeruginosa* PA14 CFU, and enhanced the expressions of putative antimicrobial genes (*K08D8.5* and *F55G11.7*) (Fig. 7). In the transgenic strain overexpressing intestinal *pmk-1*, we found that mutation of *mir-355* significantly suppressed the survival, increased the *P. aeruginosa* PA14 CFU, and inhibited the expressions of putative antimicrobial genes (*K08D8.5* and *F55G11.7*) (Fig. 7). Moreover, after *P. aeruginosa* PA14 infection, *pmk-1* mutation significantly decreased the expression of *mir-355* (Fig. S3). These results suggest that *mir-355* may act downstream of PMK-1 in the p38 MAPM signaling pathway to regulate the innate immune response to *P. aeruginosa* PA14 infection.

### Genetic interaction between *mir-355* and DAF-16 or SKN-1 in the regulation of innate immune response to *P. aeruginosa* PA14 infection

**Figure 8:**
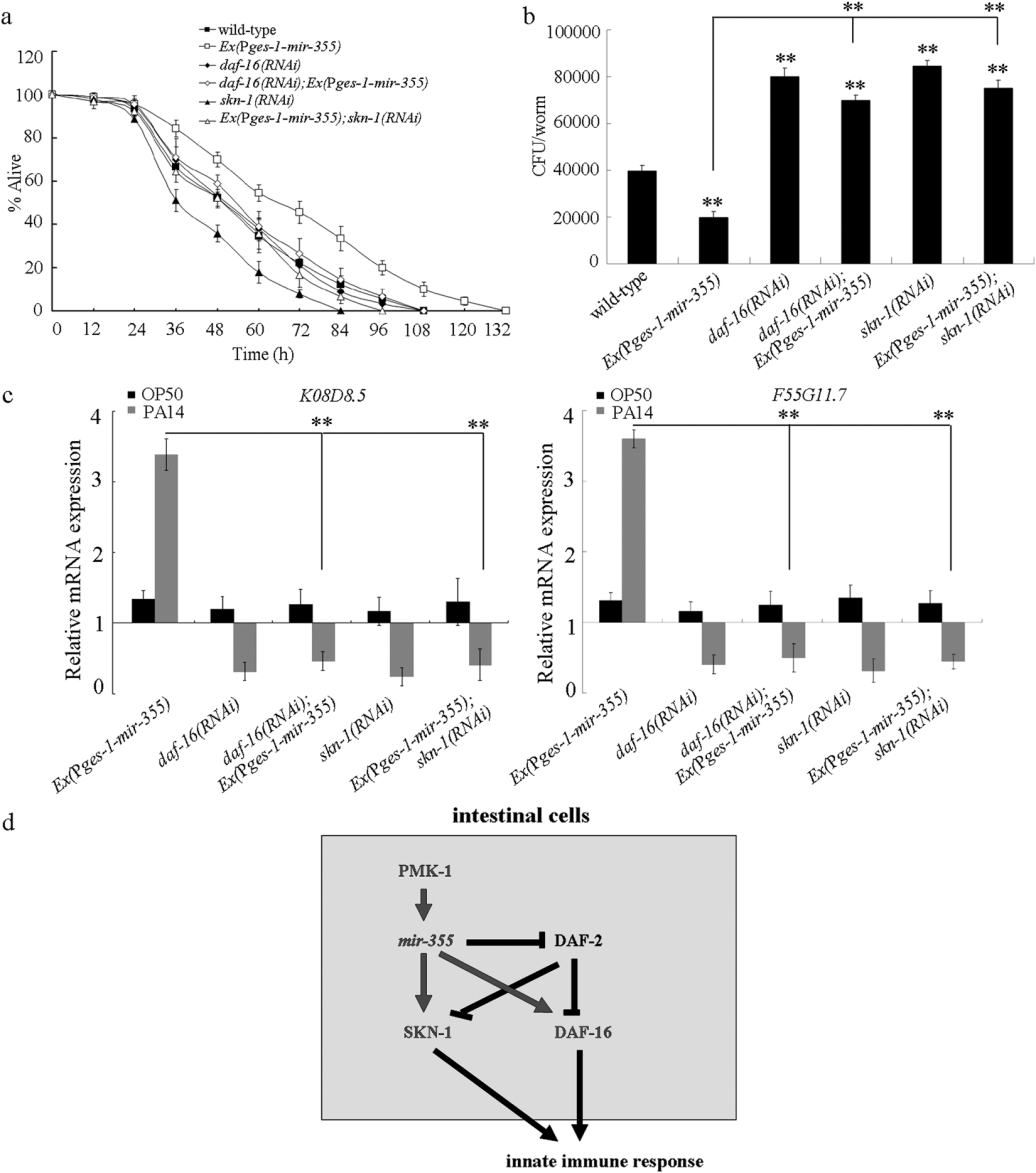
Genetic interaction between *mir-355* and DAF-16 or SKN-1 in the regulation of innate immune response to *P. aeruginosa* PA14 infection. (**a**) Genetic interaction between *mir-355* and DAF-16 or SKN-1 in the regulation of survival in *P. aeruginosa* PA14 infected nematodes. Statistical comparisons of the survival plots indicate that, after *P. aeruginosa* PA14 infection, the survival of *daf-16(RNAi);Ex(*P*ges-1-mir-355)* or *Ex(*P*ges-1-mir-355);skn-1(RNAi)* was significantly different from that of *Ex(*P*ges-1-mir-355)* (*P* < 0.001). Bars represent mean ± SD. (**b**) Genetic interaction between *mir-355* and DAF-16 or SKN-1 in the regulation of *P. aeruginosa* PA14 CFU in the body of nematodes. Bars represent mean ± SD. **P < 0.01 *vs* wild-type (if not specially indicated). (**c**) Genetic interaction between *mir-355* and DAF-16 or SKN-1 in the regulation of expression patterns of putative antimicrobial genes in *P. aeruginosa* PA14 infected nematodes. Normalized expression is presented relative to wild-type expression. Bars represent mean ± SD. **P < 0.01. (**d**) A diagram showing the molecular basis for *mir-355* in the regulation of innate immune response to *P. aeruginosa* PA14 infection.

In *C. elegans*, DAF-16, a FOXO transcriptional factor, act downstream of DAF-2 in the insulin signaling pathway to regulate the innate immune response to pathogen infection^16^. SKN-1, a bZip transcriptional factor, functions in the p38 MAPK signaling pathway to regulate diverse biological processes, such as stress response^28^. Meanwhile, SKN-1 can be directly phosphorylated by some kinases downstream of DAF-2 in the insulin signaling pathway^29^. Additionally, the activation of SKN-1 in response to pathogens is dependent on p38 MAPK signaling^30^. We found that RNA interference (RNAi) knockdown of *daf-16* or *skn-1* suppressed the survival, increased the *P. aeruginosa* PA14 CFU, and decreased the expressions of putative antimicrobial genes (*K08D8.5* and *F55G11.7*) in *P. aeruginosa* PA14 infected transgenic strain of *Ex(*P*ges-1-mir-355)* (Fig. 8a–c). After *P. aeruginosa* PA14 infection, we further found that the stain of *daf-16(mu86);Is(*P*ges-1-mir-355);skn-1(RNAi)* showed more severely suppressed survival compared with the strain of *daf-16(mu86);Is(*P*ges-1-mir-355)* or the strain of *Is(*P*ges-1-mir-355);skn-1(RNAi)* (Fig. S4).

After *P. aeruginosa* PA14 infection, *mir-355* mutation induced a significant decrease in *daf-16* expression (Fig. S5). In *C. elegans*, *skn-1* has three different isoforms. *skn-1a* and *skn-1c* are expressed in the intestine, and *skn-1b* is expressed in the neurons. After *P. aeruginosa* PA14 infection, *mir-355* mutation induced a significant decrease in *skn-1a* or *skn-1c* expression, whereas *mir-355* mutation did not significantly affect the *skn-1b* expression (Fig. S5).

## Discussion

In *C. elegans*, with the exception of *lin-4*, *let-7*, *lsy-6*, and *mir-1*, individual deletion of most of the miRNAs did not cause the overt phenotypes^31^, and the majority of miRNA may be not essential for the developmental control^32^. In contrast to these, a large amount of miRNAs were differentially expressed during the aging, and some miRNAs have been shown to be involved in the control of aging on the level of organism lifespan, tissue aging or cellular senescence in *C. elegans* ^33^. In this study, we further performed the systematic identification of possible miRNAs involved in the control of innate immune response to *P. aeruginosa* PA14 infection. Based on the phenotypic analysis of survival, we identified 11 miRNAs (*let-7*, *mir-45*, *mir-63*, *mir-75*, *mir-84*, *mir-241*, *mir-246*, *mir-256*, *mir-355*, *mir-233*, and *mir-360*) having the function in the control of *P. aeruginosa* PA14 infection (Fig. 1). Among these 11 miRNAs, *mir-45*, *mir-63*, *mir-75*, *mir-246*, *mir-256*, *mir-355*, and *mir-360* are new identified miRNAs with the function in the control of innate immunity. Among these new identified miRNA mutants, *mir-45(n4280)*, *mir-75(n4472)*, *mir-246(n4636)*, and *mir-256(n4471)* mutants were resistant to *P. aeruginosa* PA14 infection, whereas *mir-63(n4568)*, *mir-355(n4618)*, and *mir-360(n4635)* mutants were susceptible to *P. aeruginosa* PA14 infection (Fig. 1). Under normal conditions, loss-of-function mutation of *mir-45*, *mir-63*, *mir-75*, *mir-246*, *mir-256*, or *mir-355* did not obviously affect the longevity (data not shown). Under normal conditions, loss-of-function mutation of *mir-360* also does not affect the longevity^21^.

In this study, the CFU assay demonstrated that the *P. aeruginosa* PA14 infected *mir-63(n4568), mir-355(n4618)*, and *mir-360(n4635)* mutants had the enhanced *P. aeruginosa* PA14 colony formation in the body compared with *P. aeruginosa* PA14 infected wild-type nematodes; however, the *P. aeruginosa* PA14 infected *mir-45(n4280)*, *mir-75(n4472)*, *mir-246(n4636)*, and *mir-256(n4471)* mutants had the decreased *P. aeruginosa* PA14 colony formation in the body compared with *P. aeruginosa* PA14 infected wild-type nematodes (Fig. 2). These results suggest that the observed susceptibility to *P. aeruginosa* PA14 infection in *mir-63(n4568), mir-355(n4618)*, or *mir-360(n4635)* mutant may be at least partially due to the enhanced *P. aeruginosa* PA14 colony formation in the body of nematodes, and the observed resistance to *P. aeruginosa* PA14 infection in *mir-45(n4280)*, *mir-75(n4472)*, *mir-246(n4636)*, or *mir-256(n4471)* mutant may be at least partially due to the suppressed *P. aeruginosa* PA14 colony formation in the body of nematodes. Moreover, the analysis on expression patterns of putative antimicrobial genes further suggested that the observed susceptibility to *P. aeruginosa* PA14 infection in *mir-63(n4568), mir-355(n4618)*, or *mir-360(n4635)* mutant may be also largely due to the decreased expression of the examined antimicrobial genes, and the observed resistance to *P. aeruginosa* PA14 infection in *mir-45(n4280)*, *mir-75(n4472)*, *mir-246(n4636)*, or *mir-256(n4471)* mutant may be largely due to the increased expression of the examined putative antimicrobial genes (Fig. 3). Interestingly, mutations of these miRNAs induced different expression patterns of the putative antimicrobial genes in *P. aeruginosa* PA14 infected nematodes (Fig. 3), implying that the new identified 7 miRNAs may regulate the innate immune response to *P. aeruginosa* PA14 infection through different molecular mechanisms.

In *C. elegans*, *mir-45* has been shown to be involved in the control of toxicity formation of multi-walled carbon nanotubes^34^. *mir-63* was involved in the control of embryonic hypoxic response^35^. *mir-246* regulates both the longevity and the embryonic hypoxic response^35,36^. It was reported that *mir-355* could regulate the toxicity of multi-walled carbon nanotubes^34^. Besides the innate immune response to fungal infection^21^, *mir-360* has also been shown to be involved in the control of reproductive toxicity of graphene oxide and the beneficial effects of glycyrrhizic acid against the toxicity of graphene oxide^37,38^. In contrast, the biological functions of *mir-75* and *mir-256* are still unclear. In this study, our results further indicate the novel function of these 7 miRNAs in the regulation of innate immunity. After *P. aeruginosa* infection, we observed the significant increase in *mir-355* expression (Fig. S1), which implies that the *mir-355* expression may be activated to mediate a protection mechanism for nematodes against the *P. aeruginosa* infection in nematodes.

Previous studies have identified the potential target(s) for some miRNAs involved in the control of innate immunity in nematodes. For example, *mir-233* is directly targeted to SCA-1, a homologue of the sarco/endoplasmic reticulum Ca^2+^-ATPase, to regulate the innate immune response to *P. aeruginosa* infection^18^. *let-7* might be directly target to LIN-41 or to HBL-1 to regulate the innate immunity in *P. aeruginosa* infected nematodes^20^. SKN-1/Nrf could act the direct target for both *mir-84* and *mir-241*, another two members in the *let-7* family, in the control of innate immune response to *P. aeruginosa* infection^19^. With the aid of TargetScan and miRBase, we found that some of the new identified 7 miRNAs may regulate the innate immune response to *P. aeruginosa* infection by at least suppressing the functions of insulin or TGF-β signaling pathway. This information further reflects the crucial roles of insulin and TGF-β signaling pathways in the regulation of innate immune response to *P. aeruginosa* infection. Moreover, the predicted targets in insulin and TGF-β signaling pathways provide important clues for further elucidating the underlying mechanisms of new identified miRNAs in the regulation of innate immunity.

Importantly, some of the candidate miRNAs are conserved in human^39^. Among the new identified miRNAs involved in the control of innate immunity, *mir-45* is the homologue of human *miR-134* and *miR-708*, *mir-63* is the homologue of human *miR-96*, *miR-183*, *miR-200a*, and *miR-514*, *mir-75* is the homologue of human *miR-9*, *miR-320*, and *miR-548a*, and *mir-256* is the homologue of human *miR-1*, *miR-122*, *miR-206*, and *miR-519* ^39^. The data obtained in *C. elegans* imply that the homologues of these *C. elegans* miRNAs in human might be also very important for the innate immunity regulation.

In this study, based on the genetic interaction assay between *mir-355* and DAF-2 (Fig. 4), we confirmed that DAF-2 in the insulin signaling pathway may act as the potential target for *mir-355* in the regulation of innate immune response to *P. aeruginosa* PA14 infection. More importantly, the investigations on the effects of intestinal overexpression of *daf-2* lacking 3′ UTR or containing 3′ UTR on innate immunity in nematodes overexpressing intestinal *mir-355* suggested the 3′ UTR binding property of *mir-355* to DAF-2 during the control of innate immune response to *P. aeruginosa* PA14 infection (Figs 5 and 6). Our results further imply the crucial function of *mir-355*-DAF-2 signaling cascade in the intestinal cells in the regulation of innate immune response to *P. aeruginosa* PA14 infection.

Moreover, in this study, we found that *mir-355* mutation could suppress the resistance of *Ex(*P*ges-1-pmk-1)* to *P. aeruginosa* PA14 infection (Fig. 7), and RNAi knockdown of *daf-16* or *skn-1* could suppress the resistance of *Ex(*P*ges-1-mir-355)* to *P. aeruginosa* PA14 infection (Fig. 8a–c). Therefore, *mir-355* may act downstream of PMK-1 and upstream of DAF-16 or SKN-1 to regulate the innate immune response to *P. aeruginosa* PA14 infection. That is, a signaling cascade of PMK-1-*mir-355*-SKN-1 and a signaling cascade of *mir-355*-DAF-2-DAF-16 may be formed simultaneously in nematodes against the *P. aeruginosa* PA14 infection. Our results demonstrate the role of *mir-355* in linking the p38 MAPK signaling pathway and the insulin signaling pathway in the regulation of innate immune response to *P. aeruginosa* PA14 infection (Fig. 8d). Our data further provide the important molecular basis for intestinal *mir-355* in the regulation of innate immunity.

In conclusion, we performed the large scale genetic screen of miRNAs involved in the control of innate immune response to *P. aeruginosa* PA14 infection using deletion miRNA mutants. Based on this large scale deletion studies, we identified 7 novel miRNAs involved in the control of innate immune response to *P. aeruginosa* PA14 infection. Among these 7 novel miRNAs, loss-of-function mutant of *mir-45*, *mir-75*, *mir-246*, or *mir-256* was resistant to *P. aeruginosa* PA14 infection, whereas loss-of-function mutant of *mir-63, mir-355*, or *mir-360* was susceptible to *P. aeruginosa* PA14 infection. Our results proved the novel functions of these 7 miRNAs in the regulation of innate immunity. Some proteins in the insulin or TGF-β signaling pathway might act as the potential targets for these 7 miRNAs in the regulation of innate immunity. Moreover, we found that DAF-2 in the insulin signaling pathway can act as the target for *mir-355* in the intestine to regulate the innate immunity. During the control of innate immunity, *mir-355* may function as an important molecular link between the p38 MAPK signaling pathway and the insulin signaling pathway.

## Methods

### *C. elegans* strains

Nematodes strains used in the present study were wild-type N2, mutants of *let-7(mg279) X*, *lsy-6(ot71) V*, *lin-4(e912) II*, *mir-1(n4101) I, mir-2(n4108) I, mir-34(n4276) X, mir-35(gk262) II, mir-35-41(nDf50) II, mir-42-44(nDf49) II, mir-45(n4280) II, mir-46(n4475) III, mir-47(gk167) X, mir-51(n4473) IV, mir-52(n4100) IV, mir-53(n4113) IV, mir-54&55(nDf58) X, mir-57(gk175) II, mir-58(n4640) IV, mir-59(n4604) IV, mir-60(n4947) II, mir-61&250(nDf59) V, mir-62(n4539) X, mir-63(n4568) X, mir-64-66&229(nDf63) III, mir-67(n4899) III, mir-70(n4109) V, mir-71(n4115) I, mir-72(n4130) II, mir-73-74(nDf47) X, mir-75(n4472) X, mir-76(n4474) III, mir-77(n4286) II, mir-78(n4637) IV, mir-79(n4126) I, mir-80(nDf53) III, mir-81-82(nDf54) X, mir-83(n4638) IV, mir-84(n4307) X, mir-85(n4117) II, mir-86(n4607) III, mir-87(n4104) V, mir-124(n4255) IV, mir-228(n4382) IV, mir-230(n4535) X, mir-231(n4571) III, mir-232(nDf56) IV, mir-233(n4761) X, mir-234(n4520) II, mir-235(n4504) I, mir-237(n4296) X, mir-238(n4112) III, mir-239a&239b(nDf62) X, mir-240&786(n4541) X, mir-241(n4316) V, mir-242(n4605) IV, mir-243(n4759) IV, mir-244(n4367) I, mir-245(n4798) I, mir-246(n4636) IV, mir-247&797(n4505) X, mir-249(n4983) X, mir-251(n4606) X, mir-252(n4570) II, mir-253(nDf64) V, mir-254(n4470) X, mir-256(n4471) V, mir-257(n4548) V, mir-258.2(n4797) X, mir-259(n4106) V, mir-260(n4601) II, mir-261(n4594) II, mir-265(n4534) IV, mir-268(n4639) V, mir-269(n4641) IV, mir-270(n4595) IV, mir-273(n4438) I, mir-355(n4618) II, mir-357-358(nDf60) V, mir-359(n4540) X, mir-360(n4635) X*, *pmk-1(km25)IV, daf-2(e1370) III*, and *mir-355(n4618);daf-2(e1370)*, and transgenic strains of *Ex(*P*ges-1-pmk-1)*, *mir-355(n4618);Ex(*P*ges-1-pmk-1)*, *Ex(*P*ges-1-daf-2-3*′*UTR)*, *Ex(*P*ges-1-daf-2* + *3*′*UTR)*, *Is(*P*ges-1-mir-355)*, *daf-16(RNAi);Is(*P*ges-1-mir-355)*, *daf-16(mu86);Is(*P*ges-1-mir-355)*, *Is(*P*ges-1-mir-355);skn-1(RNAi)*, *daf-16(mu86);Is(*P*ges-1-mir-355);skn-1(RNAi)*, *Is(*P*ges-1-mir-355);Ex(*P*ges-1-daf-2-3*′*UTR)*, and *Is(*P*ges-1-mir-355);Ex(*P*ges-1-daf-2* + *3*′*UTR)*. *Is(*P*ges-1-mir-355)* is a transgenic strain with multi-copy *mir-355* insertion. All the used miRNA mutants are deletion mutants^8,9,31^. The mutants were backcrossed with wild-type for at least four times. In *nDf64*, *mir-253* and part of *F44E7.5* are deleted. Some of the used strains were from *Caenorhabditis* Genetics Center, which is funded by the NIH Office of Research Infrastructure Programs (P40 OD010440). Nematodes were normally maintained on nematode growth medium (NGM) plates seeded with *Escherichia coli* OP50 as a food source at 20 °C as described^40^.

### *P. aeruginosa* PA14 pathogenesis assay

Age synchronous populations of young adults were prepared, and infected with *P. aeruginosa* PA14 as described^41^. *P. aeruginosa* PA14 cultured in Luria broth was seeded on the killing plates containing a modified NGM (0.35% instead of 0.25% peptone). *P. aeruginosa* PA14 was incubated first at 37 °C for 24-h, and then at 25 °C for 24-h. *P. aeruginosa* PA14 infection was started by adding 60 young adult nematodes to the killing plates at 25 °C. Full-lawn PA14 killing plates were prepared for the *P. aeruginosa* PA14 infection.

### Survival assay

Survival assay was performed basically as described^42^. During the *P. aeruginosa* PA14 infection, nematodes were scored for dead or live every 12-h. Nematodes were counted as dead, if no response was detected after prodding with a platinum wire. Nematodes were transferred daily at 25 °C (if not specially indicated) for the first 5 days of adulthood. For the survival assay, graphs are representative of three trials. The survival curves were considered to be significantly different from the control, when the *p-*values were less than 0.001.

### Bacterial CFU assay

The CFU of *P. aeruginosa* PA14 was analyzed as described previously^43^. Young adult nematodes were infected with *P. aeruginosa* PA14 infection for 24-h. After *P. aeruginosa* infection, the examined nematodes were transferred into a M9 buffer containing 25 mM levamisole to stop pharyngeal pumping. The nematodes were placed onto a NGM plate containing ampicillin (1 mg/mL) and gentamicin (1 mg/mL) for 15-min to eliminate *P. aeruginosa* PA14 stuck onto the body surface of animals. The nematodes were transferred onto a new NGM plate containing ampicillin (1 mg/mL) and gentamicin (1 mg/mL) for 30-min to further eliminate the external *P. aeruginosa* PA14. The nematodes were lysed with a motorized pestle, and the lysates were serially diluted with M9 buffer. The diluted lysates were plated onto Luria-Bertani plates containing rifampicin (100 μg/mL) for the selection of *P. aeruginosa* PA14. After incubation at 37 °C overnight, colonies of *P. aeruginosa* PA14 were counted for the determination of CFU per nematode. Six replicates of ten nematodes each were performed.

### Quantitative real-time polymerase chain reaction (qRT-PCR)

The young adult nematodes were infected with *P. aeruginosa* PA14 for 24-h. Total RNA (~1 μg) of nematode was extracted using an RNeasy Mini kit (Qiagen), and reverse-transcribed using a cDNA Synthesis kit (Bio-Rad Laboratories). qRT-PCR was performed at an optimized annealing temperature of 58 °C. The examined putative antimicrobial genes were *lys-1*, *lys-8*, *clec-85*, *dod-22*, *K08D8.5*, *F55G11.7*, and *F55G11.4*. Relative quantification of targeted genes in comparison to the reference *tba-1* gene encoding a tubulin was determined. The expression of *mir-355* is presented as the relative expression ratio between *mir-355* and *F35C11.9*, which encodes a small nuclear RNA U6. The primer used for the transcription of *mir-355* was GTCGTATCCAGTGCAGGGTCCGAGGTATTCGCACTGGATACGAC CATAGCT. The primer for qRT-PCR of *mir-355* was TGCTAC TTTGTTTTAGCCTGAG, and the common reward primer was GTGCAGGGTCCGAGGT. The primers for qRT-PCR of *F35C11.9* were GAAGATTAGCATGAACCC and TTGGAACGCTTTATGAAT. The designed primers for targeted genes and reference *tba-1* gene were shown in Table S2. Three replicates were performed.

### RNAi assay

RNAi was basically performed by feeding nematodes with *E. coli* strain HT115 (DE3) expressing double-stranded RNA that is homologous to a targeted gene^44^. *E. coli* HT115 (DE3) grown in LB broth containing ampicillin (100 μg/mL) was plated onto NGM plants containing ampicillin (100 μg/mL) and isopropyl 1-thio-β-D-galactopyranoside (IPTG, 5 mM). L1 larvae nematodes were transferred onto RNAi plates for 2 days at 20 °C until they developed into the gravid. The gravid adults were transferred onto a fresh RNAi-expressing bacterial lawn to let them lay eggs so as to obtain the second generation of RNAi population. The eggs were allowed to develop into young adults for the subsequent assays of lifespan, CFU, and gene expression pattern.

### DNA constructs and germline transformation

To generate entry vector carrying promoter sequence, the *ges-1* promoter used for intestine-specific expression was amplified by PCR from *C. elegans* genomic DNA. The *ges-1* promoter was inserted into pPD95_77 vector in the sense orientation. The *mir-355*, *pmk-1*, and *daf-2*/*Y55D5A.5 g* cDNA lacking 3′-UTR or containing 3′-UTR were amplified by PCR, and inserted into the corresponding entry vector behind the *ges-1* promoter. Transgenic nematodes were generated as described by coinjecting testing DNA at a concentration of 10–40 μg/mL and marker DNA (P*dop-1::rfp*) at a concentration of 60 μg/mL into the gonad of nematodes^45^. To generate the transgenic strain *Is(*P*ges-1-mir-355)*, the integration of extrachromosomal array by UV irradiation was performed as described^46^. The designed primers for DNA construct generation were shown in Table S3.

### 3′-UTR reporters and microscopy

The 3′-UTR (wt) of *daf-2* was amplified by PCR from the genomic DNA. The synthesized *daf-2* 3′-UTR (mut) sequence is: ATAGAATTCTAACCCCCAAAAAATCCCGCCTCTTAAATTATAAATTATCTCCCACATTATCATATCTCTACACGAATATCGGATTTTTTTTCAGATTTTTTCTGAAAAATTCTGAATAATTTTACCCCATTTTTCAAATCTCTGTATTTTTTTTTGTTATTACCCCCCATATACATTGTGACGAGTCCTAAGACAAGAGCCCCCTTGCAACAAAAAACCATCAAAAACTTCCCGTGAATTCCATAGATAGTGTCTTTCAAACAAGATTTTTTTCTGAGTTTGTACGTTCGCTGACGAAAATTTCATGTGAAAAATTGAATTTTTGTCGATTTTTTGAGCTTAAAATCGATAATTTTTGAATTTCCCGGTAAAAAACGATAATGTATCGATTAAAAGAATGCGGGGCCCTAT. The 3′ UTR reporter construct (P*ges-1::GFP-3*′*-UTR (daf-2 wt)* or P*ges-1::GFP-3*′*-UTR (daf-2 mut)*) and mCherry internal control (*Pges-1::mCherry-3*′*-UTR (tag-196)*) plasmid were coinjected into the gonad of nematodes as described^46^. The expression of GFP and mCherry was observed and analyzed under a fluorescence microscope (Olympus BX41, Olympus Corporation, Japan). The designed primers for DNA construct generation were shown in Table S3.

### Statistical analysis

All data in this article were expressed as means ± standard deviation (SD). Graphs were generated using Microsoft Excel (Microsoft Corp., Redmond, WA). Statistical analysis was performed using SPSS 12.0 (SPSS Inc., Chicago, USA). Differences between groups were determined using analysis of variance (ANOVA). Probability levels of 0.05 and 0.01 were considered statistically significant. Lifespan was analyzed using the log-rank test.

## Electronic supplementary material

Supporting Information
