# Supplementary material for: mir-355 Functions as An Important Link between p38 MAPK Signaling and Insulin Signaling in the Regulation of Innate Immunity
Source: Sci Rep. 2017 Nov 6;7:14560. doi: 10.1038/s41598-017-15271-2 (PMC5673931; doi:10.1038/s41598-017-15271-2)
Supplement: Supplementary file 1 — Supporting Information [file 41598_2017_15271_MOESM1_ESM.doc]

***mir-355* Functions as An Important Link between p38 MAPK Signaling and Insulin Signaling in the Regulation of Innate Immunity**

Lingtong Zhi, Yonglin Yu, Zhixia Jiang & Dayong Wang^*^

Key Laboratory of Developmental Genes and Human Diseases in Ministry of Education, Medical School, Southeast University, Nanjing 210009, China

Correspondence and requests for materials should be addressed to D.W. (email: [dayongw@seu.edu.cn](mailto:dayongw@seu.edu.cn))

**Supporting Information:**

**
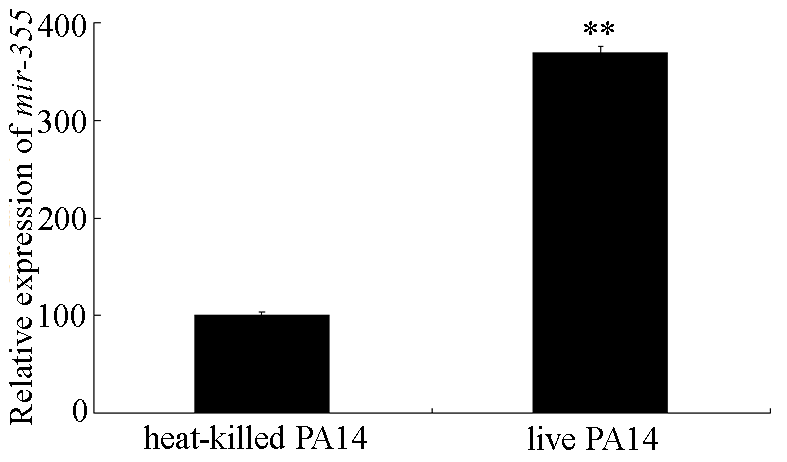
**

**Figure S1.** **Expression of *mir-355* in wild-type nematodes after *P. aeruginosa* PA14 infection for 24-h.**  Bars represent mean ± SD. ^**^*P* < 0.01 *vs* heat-killed PA14.

**
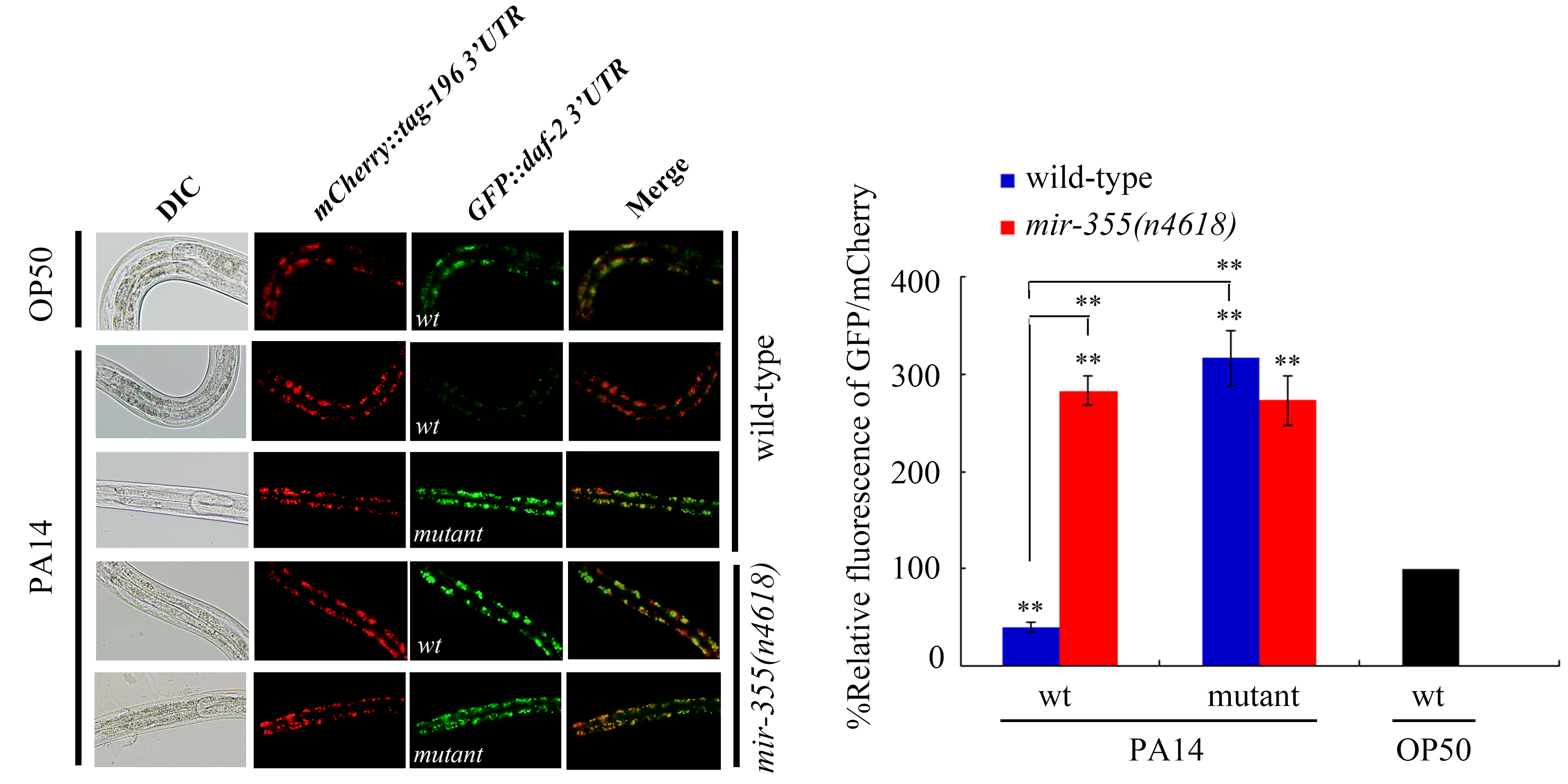
**

**Figure S2. Fluorescence images of the *daf-2*-3'-UTR GFP reporter in nematodes grown on *E*. *coli* OP50 or *P*. *aeruginosa* PA14.** Bars represent mean ± SD. ***P* < 0.01 *vs* wt on OP50 (if not specially indicated).

**
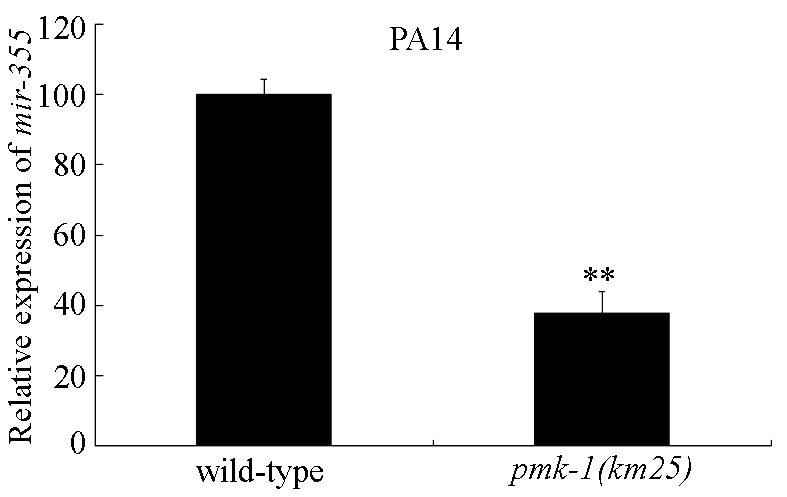
**

**Figure S3. Expression of *mir-355* in wild-type and *pmk-1* mutant nematodes after *P. aeruginosa* PA14 infection for 24-h.**  Bars represent mean ± SD. ^**^*P* < 0.01 *vs* wild-type.

**
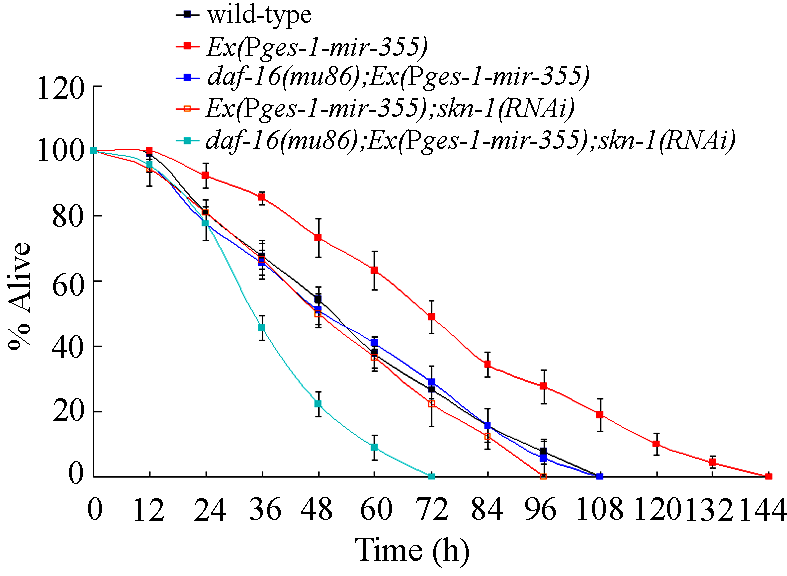
**

**Figure S4. Genetic interaction between *mir-355* and DAF-16 or SKN-1 in the regulation of survival in *P. aeruginosa* PA14 infected nematodes.** Statistical comparisons of the survival plots indicate that, after *P. aeruginosa* PA14 infection, the survival of *daf-16(mu86);Ex(*P*ges-1-mir-355);skn-1(RNAi)* was significantly different from that of *Ex(*P*ges-1-mir-355)* (*P* < 0.001). After *P. aeruginosa* PA14 infection, the survival of *daf-16(mu86);Ex(*P*ges-1-mir-355);skn-1(RNAi)* was significantly different from that of *daf-16(mu86);Ex(*P*ges-1-mir-355)* or *Ex(*P*ges-1-mir-355);skn-1(RNAi)* (*P* < 0.001). Bars represent mean ± SD.

**
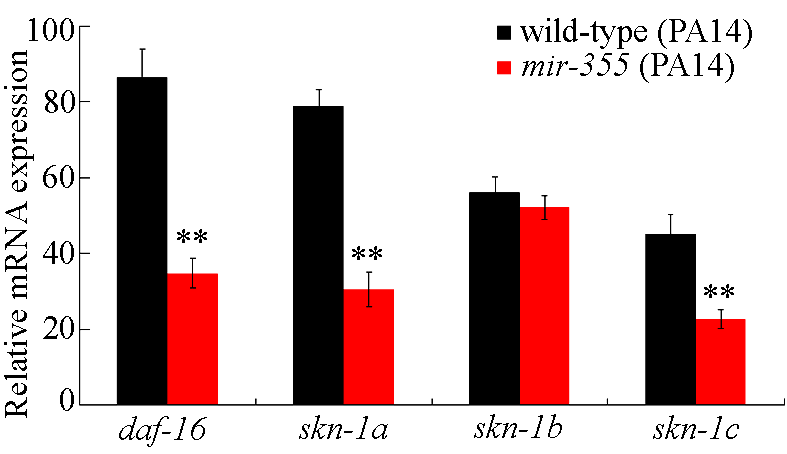
**

**Figure S5. Expression of *daf-16* and *skn-1* genes in wild-type and *mir-355* mutant nematodes after *P. aeruginosa* PA14 infection for 24-h.**  Bars represent mean ± SD. ^**^*P* < 0.01 *vs* wild-type.

**Table S1** Comparison of the survival in wild-type and miRNA mutant nematodes after *P. aeruginosa* PA14 infection

| Strains | *P* value *vs* wild-type | Strains | *P* value *vs* wild-type |
| --- | --- | --- | --- |
| *let-7(mg279)* | *P* < 0.001 | *mir-87(n4104)* | 0.8342 |
| *lsy-6(ot71)* | 0.7380 | *mir-124(n4255)* | 0.2696 |
| *lin-4(e912)* | 0.6631 | *mir-228(n4382)* | 0.7217 |
| *mir-1(n4101)* | 0.9851 | *mir-230(n4535)* | 0.2624 |
| *mir-2(n4108)* | 0.1571 | *mir-231(n4571)* | 0.2008 |
| *mir-34(n4276)* | 0.1077 | *mir-232(nDf56)* | 0.4025 |
| *mir-35(gk262)* | 0.4079 | *mir-233(n4761)* | *P* < 0.001 |
| *mir-35-41(nDf50)* | 0.6117 | *mir-234(n4520)* | 0.3233 |
| *mir-42-44(nDf49)* | 0.3064 | *mir-235(n4504)* | 0.2221 |
| *mir-45(n4280)* | *P* < 0.001 | *mir-237(n4296)* | 0.2833 |
| *mir-46(n4475)* | 0.3058 | *mir-238(n4112)* | 0.7070 |
| *mir-47(gk167)* | 0.7027 | *mir-239a&239b(nDf62)* | 0.4124 |
| *mir-51(n4473)* | 0.6363 | *mir-240&786(n4541)* | 0.8619 |
| *mir-52(n4100)* | 0.2005 | *mir-241(n4316)* | *P* < 0.001 |
| *mir-53(n4113)* | 0.0998 | *mir-242(n4605)* | 0.4202 |
| *mir-54&55(nDf58)* | 0.1427 | *mir-243(n4759)* | 0.6071 |
| *mir-57(gk175)* | 0.1003 | *mir-244(n4367)* | 0.9889 |
| *mir-58(n4640)* | 0.2177 | *mir-245(n4798)* | 0.9909 |
| *mir-59(n4604)* | 0.9588 | *mir-246(n4636)* | *P* < 0.001 |
| *mir-60(n4947)* | 0.1273 | *mir-247&797(n4505)* | 0.6161 |
| *mir-61&250(nDf59)* | 0.3660 | *mir-249(n4983)* | 0.4992 |
| *mir-62(n4539)* | 0.5430 | *mir-251(n4606)* | 0.3994 |
| *mir-63(n4568)* | *P* < 0.001 | *mir-252(n4570)* | 0.7421 |
| *mir-64-66&229(nDf63)* | 0.2276 | *mir-253(nDf64)* | 0.4691 |
| *mir-64–66,mir-229* | 0.3011 | *mir-254(n4470)* | 0.2211 |
| *mir-67(n4899)* | 0.1851 | *mir-256(n4471)* | *P* < 0.001 |
| *mir-70(n4109)* | 0.3230 | *mir-257(n4548)* | 0.2044 |
| *mir-71(n4115)* | 0.9312 | *mir-258.2(n4797)* | 0.1960 |
| *mir-72(n4130)* | 0.6105 | *mir-259(n4106)* | 0.4024 |
| *mir-73-74(nDf47)* | 0.8797 | *mir-260(n4601)* | 0.2598 |
| *mir-75(n4472)* | *P* < 0.001 | *mir-261(n4594)* | 0.4435 |
| *mir-76(n4474)* | 0.8704 | *mir-265(n4534)* | 0.9597 |
| *mir-77(n4286)* | 0.8357 | *mir-268(n4639)* | 0.4340 |
| *mir-78(n4637)* | 0.9777 | *mir-269(n4641)* | 0.7662 |
| *mir-79(n4126)* | 0.3943 | *mir-270(n4595)* | 0.9203 |
| *mir-80(nDf53)* | 0.3146 | *mir-273(n4438)* | 0.8633 |
| *mir-81-82(nDf54)* | 0.7549 | *mir-355(n4618)* | *P* < 0.001 |
| *mir-83(n4638)* | 0.7654 | *mir-357-358(nDf60)* | 0.9398 |
| *mir-84(n4307)* | *P* < 0.001 | *mir-359(n4540)* | 0.3427 |
| *mir-85(n4117)* | 0.3644 | *mir-360(n4635)* | *P* < 0.001 |
| *mir-86(n4607)* | 0.3789 |  |  |

**Table S2** Primers used for quantitative real-time polymerase chain reaction (PCR)

| Gene | Forward primer (5’-3’) | Reverse primer (5’-3’) |
| --- | --- | --- |
| *tba-1* | TCAACACTGCCATCGCCGCC | TCCAAGCGAGACCAGGCTTCAG |
| *lys-1* | TTCGGATCTTTCAAGAAG | TGGGATTCCAACAACGTA |
| *lys-8* | TCAGTCTCCGTCAAGGTC | GAAGCTGGCTCAATGAAA |
| *clec-85* | GGTTTTGGCTGTAGCACG | GGTTTTGGCTGTAGCACG |
| *dod-22* | CCAGGATACAGAATACGT | CCAGAGATGACTTCAGTT |
| *K08D8.5* | TTACGATGGTGATTCCGT | GCTTGTTGCCAGTTGAGA |
| *F55G11.7* | CACCCTCAGGCCAACTCA | CTGTGACTGTAGCGTCAC |
| *F55G11.4* | GGATCCGTGTATTTGGCT | GTGAAGACATATGTGCTC |
| *daf-16* | ATTCCTTCCTGGCTTTGC | CGTTTCCTTCGGATTTCA |
| *skn-1a* | AGTGCTTCTCTTCGGTAGCCG | TGTTGGACGATGGTGAACTGA |
| *skn-1b* | TGCCTCCTCTCTTCTGGCAT | GATGATGGCCGTGTTGATCC |
| *skn-1c* | CAACGGATGATGGAGTATTT | GGTTGGACGTTCTGTAGATG |

**Table S3** Primers for DNA constructs

| Gene | Forward primer (5’-3’) | Reverse primer (5’-3’) |
| --- | --- | --- |
| P*ges-1* | ATATCTAGAAGCCACTCAGCCACTTCA | ATAGGATCCCATCTGAATTCAAAGATA |
| *mir-355* | TATGGATCCTGATAAAACGTCGGCTGC | TCGCCCGGGAGATACAACACTATTCAG |
| *pmk-1* | TACCCATGGATGTTTCCACAGACAACAAT | ACTCTCGAGCTACGATTCCATTTTCTCCT |
| *daf-2* lacking 3’ UTR | ATACCCGGGATGACGCCAGGCTTCTTC | GCGGGTACCTCAGACAAGTGGATGATG |
| *daf-2* containing 3’ UTR | ATACCCGGGATGACGCCAGGCTTCTTC | GCCGGTACCGCAAAAAGAAAATCGATA |
| *daf-2* 3’ UTR (wt) | ATACCCGGGATGACGCCAGGCTTCTTC | GCCGGTACCGCAAAAAGAAAATCGATA |
| *tag-196* 3’ UTR | CCGGAATTCTCTGCACTTGTTAACTGA | TCACCCGGGCTAAGTACATACAAACTT |
